# Menstrual hygiene management and school absenteeism among female adolescent students in Northeast Ethiopia

**DOI:** 10.1186/1471-2458-14-1118

**Published:** 2014-10-29

**Authors:** Teketo Kassaw Tegegne, Mitike Molla Sisay

**Affiliations:** Department of Public Health, College of Medicine and Health Sciences, Debre Markos University, Debre Markos, Ethiopia; School of Public Health, College of Health Sciences, Addis Ababa University, Addis Ababa, Ethiopia

**Keywords:** Menstrual hygiene, Menstrual knowledge, Menarche, Sanitary napkins, Adolescent school girls, School absenteeism, School dropout

## Abstract

**Background:**

Adolescence in girls has been recognized as a special period marked with the onset of menarche. Even though menstruation is a natural process, it is associated with misconceptions, malpractices and challenges among girls in developing countries. However, much is not documented; school-absenteeism and dropout are a common problem among girls in rural Ethiopia. Focusing among school girls, this study has examined knowledge about menstruation, determinants of menstrual management and its influence on school-attendance in Northeast Ethiopia.

**Methods:**

We conducted a mixed-method research combining quantitative and qualitative methods in Northeast Ethiopia. The quantitative study was conducted among 595 randomly selected adolescent school girls. Nine in-depth interviews; five school-dropout girls and four female teachers, and four focus group discussions among school girls were conducted in 2013.

**Results:**

The mean age at menarche was 13.98 (±1.17) years. About 51% of girls had knowledge about menstruation and its management. Only a third of the girls used sanitary napkins as menstrual absorbent during their last menstruation. Girls from urban areas, had mothers of secondary and above education and, families of higher monthly expenditure had more chance of using sanitary napkins than their counterparts. More than half of the girls reported to have been absent from school during their menstruation period. Those who did not use sanitary napkins were more likely to be absent from school [AOR-95% C.I: 5.37 (3.02 - 9.55)]. Fifty eight percent of girls reported that their school-performance had declined after they had menarche. In addition, the qualitative study indicated that school-dropout was common among girls who experienced teasing and humiliation by classmates when their clothes were stained with blood as they do not use sanitary napkins.

**Conclusion:**

Though there is an effort to increase girls’ school enrollment, lack of basic needs, like sanitary napkins that facilitate routine activates of girls at early adolescence are observed to deter girls’ school-attendance in rural Ethiopia. Special support for girl students, especially when they have their first menstruation and separate functioning sanitary facilities are necessities that should be in school at all times if gender equality and girls empowerment is to be achieved.

## Background

Adolescence in girls has been recognized as a special period in their life cycle that requires due attention. This period is marked by the onset of menarche [1]. Menstruation; is unique to females and is part of the female reproductive cycle that starts at puberty [2–4]. Even though menstruation is a natural process, it is linked with several misconceptions and malpractices which may result in adverse health outcomes. Poor hygiene during menstruation has been associated with serious ill-health, including reproductive tract and urinary tract infections [3, 4].

During menarche, girls experience different feelings including fear, shame and guilt because of lack of prior information about menstruation [5]. A study done among Nigerian secondary school girls revealed that adolescent girls gave different meanings to menstruation and perceived it as physiological process, as an assurance of fecundity, and as a release of ‘bad blood’ [6]. In another study it was viewed as an event that happens to girls during puberty occurring monthly where the body gets rid of spoiled blood. However, girls who had information about menstruation before menarche had a positive attitude [7].

According to estimates of the United Nations Children’s Fund (UNICEF), about one in ten school-age African girl didn’t attend school during menstruation or dropped out at puberty due to lack of cleanliness and separate toilet facilities for female students at schools [8]. A study done in Kenya showed that the girls had faced difficulty to manage their menstrual periods at school due to lack of adequate privacy and sanitary facilities. In some schools which did not have waste disposal facilities, girls were obliged to carry soiled absorbents back home. As a result girls preferred to stay at home during their menstruation period [9].

Several studies documented that menstruation related problems, had affected more than a third of student’s class concentration, participation, socializing with friends, test-taking skills and homework task performance. Dysmenorrhea was significantly associated with school absenteeism and decreased academic performance, sports participation, and socialization with peers [10, 11].

A study conducted in Ethiopia showed that, though, most (92%) students were aware of menstruation before menarche, their utilization of sanitary napkins was low at 37.6% and a significant proportion, 62.4% were using rags and pieces of cloth [12]. Urban–rural disparity in access to sanitary napkins indicated that, 37.1% of urban girls used sanitary napkins while only 1.6% of rural girls used this product. Sanitary napkins use was limited due to access and financial constraints [13].

Due to menstruation related problems, 43% - 50.7% of students were absent from school, ranging from one day to four days [12, 14, 15]. About 90% of students reported that their school did not have a separate sanitary facility for females and about 43% of informants were obliged to have missed school during their menstruation days [14]. Lacks of separate facilities were also related with a high rate of female school dropouts in Oromia (65%) and Amhara (33%) regional states of Ethiopia [16].

Moreover, students had a difficulty of attending class attentively due to menstrual related problems such as pain and fear of sudden menstrual blood leakage, as they did not use proper sanitary napkins [15]. About 39% of respondents perceived that menstruation had affected their academic performance or rank negatively when compared to their rank before menarche. They also had discomfort and shame sitting besides male students in the class [15].

Apart from proper school attendance, most girls in rural Ethiopia are at risk of getting genitourinary tract infections due to their unhygienic practices mentioned above during their menstruation period which will lead to further complication if left untreated [13].

Ethiopia is a poor country where more than 80% of the population is residing in rural areas [17]. Problems encountered by girl students in Ethiopia with regard to menstruation management, especially poor school attendance and academic performance as well as school dropout may seriously hamper the realization of the Millennium Development Goals (MDG-2) on universal education and MDG-3 on gender equality and women empowerment.

However, much attention is not given to this problem and studies on menstruation and its hygienic management as well as its influence on girls’ education are limited and scarce in Ethiopia. This study is therefore conducted with the aim of assessing the prevailing knowledge about menstruation and its hygienic management, identifying factors that affect hygienic management of menstruation and to assessing the associated consequences of menstruation related problems on school attendance and dropout among adolescent school girls.

## Methods

### Study area

Habru is one of the woredas in North Wollo zone, located at a distance of 490 km to the Northeast of Addis Ababa in Amhara Regional State of Ethiopia. The Woreda has a total population of 192,742 where 96,874 were men and 95,868 women. Only 21,600 (11.21%) were urban inhabitants and the majority of the inhabitants (77%) were Muslims [17]. The Woreda had a total of 98 public primary schools. Of the total number of 31,361students enrolled in primary education in the year 2012/13; 15,642 were male and 15,719 were females [18].

### Study design

We conducted a mixed-method research by combining a cross-sectional survey and qualitative research. The survey was conducted among female adolescent students. In-depth interviews were conducted among school dropout girls and female teachers while focus group discussions were conducted among school girls. The interviews and focus group discussions explored female students and school dropout girls’ views about menstruation and its hygienic management and influence of menstruation on girls’ academic performance, school absenteeism and school dropout. In addition, we wanted to capture female teachers’ perceptions and experiences of their students’ menstrual knowledge, hygienic management, school sanitation facilities and influence of menstruation on girls’ education.

### Sampling

#### Quantitative study

The sample size was calculated using three scenarios for computing sample size: (1) using the prevalence of knowledge about menstruation at 92% [12], (2) the prevalence of sanitary napkins use at 37.6% [12] and (3) the prevalence of school-absenteeism at 17% [13]. Based on these assumptions the 37.6% prevalence was taken which gave us a larger sample size. Assuming a 95% confidence level, and the acceptable difference of 5% we found a sample size of 361. Using a design effect of 1.5 and 10% non-response rate, the final sample size became 595.

The students were selected using a multistage sampling technique: 36 schools having grades, 7th and 8th out of 98 primary schools were selected on stage one. At stage two, seven schools having grades 7th and 8th were randomly chosen from the 36 schools using simple random sampling. Then, at stage three, 595 students (with an age range of 10 – 19 years) were selected using a stratified random sampling method with proportionate allocation to size using list of female students (sampling frame). The strata were based on grades and sections for those schools having two or more sections for each grade.

All girl students who attended regular school were included in the study. Students who had sight problems and having hearing difficulty were not included in the quantitative part of this study.

#### Qualitative study

We conducted five in-depth interviews among adolescent girls who dropped out of school and four in-depth interviews among female teachers and four focus group discussions among female adolescent students who had their menarche. Data saturation was the marker of the sample adequacy.

The study participants from different school clubs who were enrolled in grade 7 and grade 8 were purposively selected for focus group discussions. Girls who dropped out of school and female teachers who were serving as a school counselor or those who work closely with girls were purposively selected for in-depth interviews. The selection of the participants of both dropout girls and focus group discussants; who had their menarche, was facilitated by female teachers who were consulted by students.

### Data collection

#### Quantitative study

A self-administered pre-tested close ended Amharic questionnaire was used. The questionnaire mainly focused on socio-demographic factors, parental factors, knowledge and hygienic management regarding menstruation, and problems associated with menstruation. The participants were briefed about the purpose of the study and data were collected after a written informed consent. For those students who were under the age of consent, informed verbal assent was obtained from their parents. The data collection process was facilitated by four female data collectors with health background and one supervisor. Students were instructed on how to fill the questionnaire and the data collection took an average of 40 – 60 minutes.

Data quality was assured through careful design of the questionnaire. Data collectors were trained for two days about the purpose of the study, including the rights of study subjects and the content of the questionnaire in detail as well. Data were checked for completeness and consistency after each day of data collection by holding a meeting with the data collectors.

#### Qualitative study

The study participants were informed briefly about the purpose of the study and a written informed consent was taken. Two days of training were given to the qualitative data collectors. Data were collected by female, public health professionals who had experience in counseling and a note taker and voice recorder. The qualitative data were collected in the quietest corner of the school compound that gives optimum privacy and the interviews took 45 – 90 minutes.

The interview and focus group discussion guide for students and dropout girls focused on girls’ knowledge and perception about menstruation, hygienic management practices and menstrual influence on their academic performance, school absenteeism and school dropout. The interview guide of female teachers focused on their students’ knowledge and hygienic management of menstruation, sanitary conditions of schools, menstrual influence on their students’ academic performance, school absenteeism and school dropout and their specific experiences in this regard. All information was recorded using a digital voice recorder and note was taken. The recorded data were transcribed in Amharic (local language) and then translated into English.

### Data analysis

#### Analysis and measurement of the quantitative data

Data were entered using Epi Info version 3.5.3 and analysis was performed using SPSS version 16.0 statistical software. Bivariate and multivariate models were run to assess any relationship between each independent variable (socio-demographic factors, school environment, parental or family factors, knowledge about menstruation and its hygienic management and disposable sanitary napkins use) and outcome variables (disposable sanitary napkins use and absenteeism from school). Crude and adjusted odds ratios were used to ascertain effect sizes for any association between the dependent and predictor variables while significance was determined using 95% confidence intervals. Independent variables found to be significant with p-value less than 0.05 at the bivariate level were included in a multivariate logistic regression model for the dependent variable to control potential confounding variables.

### Measurement

Knowledge of menstruation and its hygienic management was measured among those who reported they had heard about menstruation including its management. The students’ knowledge of menstruation and its hygienic management was scored using a scoring system adopted from past study [19]. Students’ menstrual knowledge score was calculated out of the 12 knowledge specific questions (see Table 1). Each correct response earned one point, whereas any wrong or don’t know response attracted no mark and thus the sum score of knowledge was calculated (12 points). The mean score of menstrual knowledge (6.95 ± 2.03) was used to decide cutoffs of the rank. Respondents that scored 0–3 points were adjudged as having poor knowledge; whereas those that scored 4–6 and 7–12 points were adjudged as having fair and good knowledge respectively (Table 2).

**Table 1:** **Adolescent school girls knowledge about menstruation and its management, Northeast Ethiopia, 2013 (n = 551)**

Variable	Numbers	Percent
Heard about menstruation before menarche	478	86.75
Feel comfortable to talk about menstruation	154	27.95
Knew common age range of menarche	501	90.93
Knew normal menstrual bleeding duration	516	93.65
Knew duration of a normal menstrual cycle	535	97.10
Knew disposable sanitary pad as menstrual soak up	262	47.55
Aware that menstruation is a physiologic process	319	57.89
Aware that menstruation is due to hormones	46	8.35
Aware that menstrual blood is from uterus	127	23.05
Aware that a girl cannot conceive during menstruation	279	50.64
Had learnt menstruation & its hygienic management in school	135	24.50
Aware that a girl can go to school during menstruation	476	86.39

NB: Multiple responses were possible and the percent is greater than 100%.

**Table 2:** **School girls knowledge grading on menstruation and its management, Northeast Ethiopia, 2013 (n = 551)**

Grading		Frequency	Percent
*Knowledge (n = 551)	Poor (0–3 points)	17	3.09
	Fair (4–6 points)	251	45.55
	Good (7–12 points)	283	51.36

NB: *Only 551 were included in knowledge scoring (23 of them didn’t heard about menstruation and didn’t start menstruation).

### Analysis of the qualitative data

The qualitative data from the interviews with dropout adolescent girls, female teachers, and focus group discussions with adolescent school girls were analyzed using an inductive content analysis [20]. To facilitate the coding process, the analysis was started by importing the translated text into the OpenCode software version 3.6 [21]. Units of relevant meaning were coded segment by segment. Two coders (the investigators) did the coding process as well as assigning the emerged categories independently. The coding and emerged category results were discussed by the investigators and discrepancies were negotiated. As part of the analysis five categories were developed that illustrated the manifest meaning of the findings, while the single theme represents the overall interpretation of the qualitative information and reflects the latent meaning of the data (Table 3).

**Table 3:** **The theme, categories and codes as identified from the qualitative data**

Theme: ‘Girl students academic performance and school attendance is affected by lack of knowledge, sanitary materials and lack of facilities at schools’					
Categories	1-Poor knowledge about menstruation	2-Unreadiness during menstruation	3-Influence on academic performance	4-Reasons of school absenteeism	5- Reasons of school dropout
Codes	No prior knowledge about menstruation	Afraid to use sanitary pad in school	Class concentration	Lack of school facilities	Fear of sudden bleeding
	Misperceptions	Knowledge gap on how to use sanitary pad	Class participation	Fear of unexpected bleeding	Lack of material or pad
	Shocked	Ashamed to buy sanitary pad	School absenteeism	Lack of material or pad	Lack of knowledge
	Scared	Unavailability of sanitary pad	School dropout	Embarrassment	Embarrassment
	Disclosure	Malpractices	Missing exam	Pain	
		High cost			

### Ethical consideration

Ethical approval was obtained from, School of Public Health, College of Health Sciences, Addis Ababa University Research Ethics Committee [22] and approval letter was obtained from Habru Woreda Education Office in the respective schools included in this study. School directors and directresses were briefed on the objectives of the study and permission to conduct the study was obtained from participating schools. The purpose of the study was explained to the students and written informed consent was obtained from the participants both for the quantitative and qualitative studies. For those students who were under the age of consent, informed verbal assent was obtained from the parents of study participants. Confidentiality of information was maintained by omitting any personal identifier from the questionnaires. The recorded data were stored in a safe place where no one except the principal investigators has access.

## Result

This paper presents the results of the survey complemented with the findings from the qualitative study using five categories representing the meaning of the findings, including *1) Poor knowledge about menstruation; 2) Unreadiness during menstruation; 3) Influence of menstruation on girls’ academic performance; 4) Reasons of school absenteeism and 5) Reasons of school dropout* (see Table 3). Quotes that illustrate how the interpretations are grounded in the data are presented.

### Socio-demographic and economic characteristics of adolescent school girls

In this study, 574 students participated obtaining a response rate of 96.47%. Most, 562 (97.91%) of them were from the Amhara ethnic group and 313 (54.53%) were Orthodox Christians. More than half, 305 (53.14%) of them were grade 7 students and 315 (54.88%) were from rural areas. Their age ranges between 12 and 19 years with a mean age of 14.96 (±1.33) years and most of them 306 (53.31%) were in mid adolescence. Mothers of most school girls were illiterate, 244 (42.51%) and were not gainfully employed (housewives), 361 (62.89%) and a third of their fathers can only read and write, 210 (36.59%) and most were farmers, 381 (66.38%) (Table 4).

**Table 4:** **Socio-demographic characteristics of adolescent school girls in Northeast Ethiopia, 2013**

Characteristics of respondents (n = 574)		Numbers	Percent
**Age group (years)**	10-13	76	13.24
	14-16	306	53.31
	17-19	192	33.45
**Religion**	Orthodox	313	54.53
	Islam	239	41.64
	Others*	22	3.83
**Residence**	Rural	315	54.88
	Urban	259	45.12
**Grade**	Grade 7	305	53.14
	Grade 8	269	46.86
**Marital status**	Never married	568	98.95
	Married	6	1.05
**Ethnicity**	Amhara	562	97.91
	Tigre	12	2.09
**Live with**	Both parents	425	74.04
	Mother only	81	14.11
	Relatives	43	7.49
	Others**	25	4.36
**Mother’s education**	Illiterate	244	42.51
	Read & write	181	31.53
	Primary	83	14.46
	Secondary and above	66	11.50
**Father’s education**	Illiterate	153	26.66
	Read & write	210	36.59
	Primary	98	17.07
	Secondary and above	113	19.69
**Mother’s occupation**	House wife	361	62.89
	Petty trader	157	27.35
	Employed*****	37	6.45
	Others***	19	3.31
**Father’s occupation**	Farmer	381	66.38
	Petty trader	81	14.11
	Employed*****	101	17.59
	Others****	11	1.92
**Monthly expenditure (Eth Birr)**	<=300	129	22.47
	301-600	170	29.62
	601-900	91	15.85
	901-1200	103	17.94
	>1200	81	14.11

NB: *(protestant and catholic), **(live with husband and father), ***(farmer, daily laborer), ****(carpenter, barber, daily laborer), *****employed (government and private).

### Adolescent school girls’ knowledge about menstruation and its hygienic management

**Figure 1:**
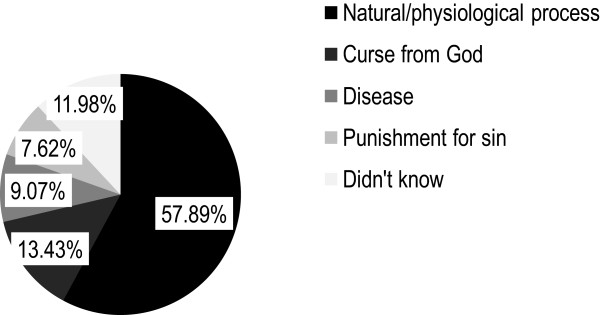
**Pie chart showing adolescent school girls knowledge about menstruation, Northeast Ethiopia, 2013 (n = 551).**

The majority of the girls, 478 (86.75%) had heard about menstruation before they had menarche (Table 1); where the leading sources of information were sisters, 204 (42.68%), followed by mothers, 183 (38.28%), friends 141 (29.50%) and teachers, 64 (13.39%). Regarding knowledge about menstruation; 319 (57.89%) of them knew correctly that menstruation is a physiologic process (Figure 1). The mean score of the school girls’ knowledge of menstruation and its hygienic management was 6.95 ± 2.03 on a scale of 1–12 (Table 2). Even though half of them; 283 (51.36%), had good knowledge about menstruation (Table 2), but there is a knowledge gap in specific areas, i.e., only 46 (8.35%) and 127 (23.05%) of them knew exactly as menstruation is due to hormones and the menstrual bleeding is from the uterus, respectively (Table 1).

Out of 574 respondents, 104 (18.12%) of them had a discussion within the family (their mothers and elderly sisters), about sexual and reproductive health (SRH) issues. Out of those who discuss SRH issues, only 31 (29.81%) of them had a discussion about menstruation and its hygienic management.

**Figure 2:**
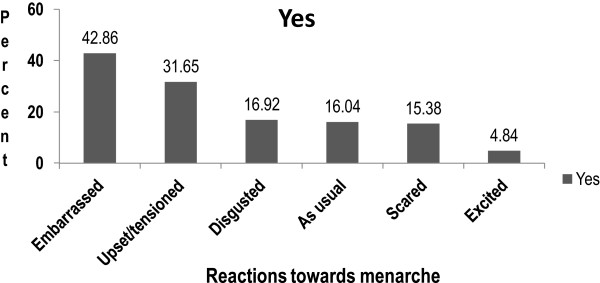
**Showing adolescent school girls reactions to menarche, Northeast Ethiopia, 2013 (n = 455).** *NB: Multiple responses were possible and the percent is greater than 100%.*

Most female students, 455 (79.27%) had their menarche where the mean age at menarche was 13.98 (±1.17) years. During the onset of menarche girls reported different feelings such as: embarrassment among 195 (42.86%) of the girls, 144 (31.65%) were upset and tensioned and 77 (16.92%) were irritated or disgusted (Figure 2). The qualitative study supports the findings from the survey as presented below.

**1-Poor knowledge about menstruation (see Table** 3**: categories)** The focus group discussions and in-depth interviews revealed that most girls had no prior knowledge about menstruation and its management. As a result they reported to have been faced with different psychological and emotional problems, including being shocked, scared and felt ashamed. They also reported that they were reserved to communicate issues (having menstruation) with anybody. Misconceptions about menstruation, such as being dirty and considering it as unhealthy situation, were common.

*“I was attending class when I had my first menstruation.... My cloth was stained with blood as I was not ready and had only underwear’s (no sanitary napkins). I went home running ahead of the students’ in order not to be seen by anyone. At home, I was afraid of being noticed by my family; I thought it would offend them and I changed my clothes without them noticing it” (****FGD participant)***

*“During my first menstruation I was shocked and embarrassed. Generally, whenever I have it, I think that I am below humans, depressed, eh … I hate being female; I assumed it as a disease.....”****(IDI- school-dropout girl)***

### Menstruation and its hygienic management

**Figure 3:**
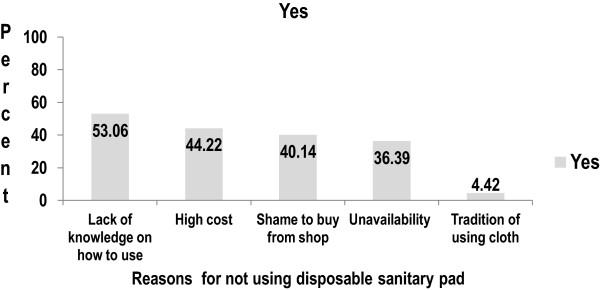
**Showing school girls reasons for not using sanitary pad, Northeast Ethiopia, 2013 (n = 294).** *NB: Multiple responses were possible and the percent is greater than 100%.*

Only 161 (35.38%) of students used sanitary napkins and the rest 253 (55.60%) and 41 (9.01%) of them used homemade cloth and underwear as menstrual soak-up during their last menstrual period respectively. Among 294 students who had used soak-ups other than sanitary napkins, 270 (91.84%) of them reused the material. The main reasons for not using sanitary napkins were lack of knowledge on how to use, 156 (53.06%), followed by high cost, 130 (44.22%), and shame to buy from shop, 118 (40.14%) (Figure 3). Two hundred sixty five (58.24%) and 95 (20.88%) of girls reported that they changed their menstrual soak up twice and more than twice a day respectively. One hundred (37.04%) of the girls washed the reusable cloth with soap and water and dried it inside the house where sunrays are not coming, 90 (33.33%).

Only 395 girls reported to have taken a bath during their menstrual period; where above half of them 217 (54.94%) responded that they washed their body when they finished their menstrual period. Out of 455 students who had their menarche, 389 (85.49%) of them did not change menstrual soak up at school. The main reasons mentioned by 177 (45.50%) students was absence of separate toilet for female students while 152 (39.07%) reported fear of other students, 73 (18.77%) reported lack of water sources, and the rest 59 (15.17%), reported shortage of sanitary napkins or material used as absorbent as a reason. Regarding the method of disposal of the used material at school, home and/or everywhere; 353 (77.58%) girls disposed the cloth pieces or sanitary napkins used into latrines and 152 (33.41%) of them in the open field (Table 5).

**Table 5:** **Adolescent school girls’ hygienic practices during menstruation, Northeast Ethiopia, 2013**

Hygienic practices		Number	Percent
**Material used during last menstrual period (n = 455)**	Disposable sanitary pad	161	35.38
	Homemade cloth	253	55.60
	Underwear	41	9.01
**Genital cleaning material (n = 319)**	Soap and water	135	42.32
	Water only	123	38.56
	Plain paper	55	17.24
	Others*	6	1.88
**Bath frequency (n = 395)**	Daily	54	13.67
	First day	21	5.32
	Second day	41	10.38
	Third day	59	14.94
	When finished period	217	54.94
	As per convenience	3	0.76
**Bath using (n = 395)**	With soap and water	348	88.10
	With water only	47	11.90
**Disposal of menstrual material used (n = 455)**	Open field	152	33.41
	Latrine	353	77.58
	Waste bins	16	3.52
**Absorbent material change per day (n = 455)**	Once	95	20.88
	Twice	265	58.24
	More than two times	95	20.88
**Drying of washed reusable cloth (n = 270)**	In the shade outside	27	9.96
	In the shade inside	90	33.33
	In the sunlight inside	29	10.74
	In the sunlight outside	27	9.96
	Hidden under other clothes	51	18.89
	Hidden elsewhere	46	17.04
**Washing of the reusable cloth (n = 270)**	With soap and water	100	37.04
	With water only	165	61.11
	Others**	5	1.85
**Place of store between use (n = 270)**	In plastic bag separately	142	52.59
	With other clothes	98	36.30
	In the bath room	30	11.11

*(toilet tissue paper, piece of cloth), **(ash, indod).

NB: Multiple responses were possible and the percent might be greater than 100% and the frequencies and percentages were calculated among those who had seen menstruation (455).

According to the multivariate analysis students who were living in urban areas were 2.32 times more likely to use a disposable sanitary napkins than their counterparts [AOR (95% C.I) 2.32 (1.21 - 4.45)]. Girls from literate families; i.e., who can read and write, completed primary and secondary education [AOR (95% C.I): 2.30 (1.23 - 4.30), 4.13 (1.85 - 9.18), 4.26 (1.61 - 11.28)] respectively were more likely to use sanitary napkins than their counterparts who were from illiterate family. Girls from families having household monthly expenditure of 601–900, 901–1200 and greater than 1200 Ethiopian Birr [AOR (95% C.I): 3.24 (1.46 - 7.17), 3.41 (1.56 - 7.43), 4.97 (2.21 - 11.16)] respectively were more likely to use sanitary napkins than their counterparts expending less than 600 Ethiopian Birr. School girls who were living with relatives [AOR (95% C.I): 0.16 (0.04 - 0.56) were less likely to use disposable sanitary napkins than their counterparts (Table 6). However, father’s education, mother’s and father’s occupation and the school environment were confounders of disposable sanitary napkins use.

**Table 6:** **Factors associated with sanitary pad use during menstruation, Northeast Ethiopia, 2013 (n = 455)**

Variables		Disposable sanitary napkin use		Crude odds ratio (95% C.I.)	Adjusted odds ratio (95% C.I.)
		No	Yes		
		n (%)	n (%)		
School	Mersa	56 (53.80)	48 (46.20)	1.00	1.00
	Sirinka	42 (65.60)	22 (34.40)	0.61 (0.32 - 1.16)	2.30 (0.99 - 5.32)
	Kokono	27 (77.10)	8 (22.90)	0.35 (0.14 - 0.83)*	0.88 (0.28 - 2.76)
	Merto	48 (72.70)	18 (27.30)	0.44 (0.23 - 0.85)*	0.83 (0.36 - 1.96)
	Sirinka Gerado	30 (73.20)	11 (26.80)	0.43 (0.19 - 0.94)*	1.82 (0.65 - 5.08)
	Mehal Amba	31 (77.50)	9 (22.50)	0.34 (0.15 - 0.78)*	0.95 (0.34 - 2.65)
	Melka Chefe	60 (57.10)	45 (42.90)	0.88 (0.51 - 1.51)	0.91 (0.48 - 1.72)
Residence	Rural	178 (76.40)	55 (23.60)	1.00	1.00
	Urban	116 (52.30)	106 (47.70)	2.96 (1.98 - 4.42)***	2.32 (1.21 - 4.45)*
Live with	Both parents	206 (60.60)	134 (39.40)	1.00	1.00
	Mother only	45 (71.40)	18 (28.60)	0.62 (0.34 - 1.12)	0.52 (0.26 - 1.03)
	Father only	9 (81.80)	2 (18.20)	0.34 (0.07 -1.61)	0.89 (0.16 - 4.82)
	Relatives	32 (91.40)	3 (8.60)	0.14 (0.04 - 0.48)**	0.16 (0.04 - 0.56)**
	Husband	2 (33.30)	4 (66.70)	3.08 (0.56 -17.02)	4.71 (0.66 - 33.63)
Mother’s education	Illiterate	159 (81.50)	36 (18.50)	1.00	1.00
	Read & write	82 (60.70)	53 (39.30)	2.85 (1.73 - 4.71)***	2.30 (1.23 - 4.30)**
	Primary	30 (44.80)	37 (55.20)	5.45 (2.98 - 9.95)***	4.13 (1.85 - 9.18)**
	Secondary and above	23 (39.70)	35 (60.30)	6.72 (3.55 - 12.73)***	4.26 (1.61 - 11.28)**
Father’s education	Illiterate	94 (76.40)	29 (23.60)	1.00	1.00
	Read & write	101 (68.20)	47 (31.80)	1.51 (0.88 - 2.59)	0.58 (0.29 - 1.15)
	Primary	49 (59.00)	34 (41.00)	2.25 (1.23 - 4.11)**	0.71 (0.32 - 1.59)
	Secondary and above	50 (49.50)	51 (50.50)	3.31 (1.87 - 5.85)***	0.71 (0.27 - 1.91)
Mother’s occupation	House wife	193 (69.20)	86 (30.80)	1.00	1.00
	Petty trader	79 (59.80)	53 (40.20)	1.51 (0.98 - 2.32)	0.94 (0.53 - 1.65)
	Employed^†††^	13 (37.10)	22 (62.90)	3.80 (1.83 - 7.89)***	1.97 (0.73 - 5.28)
	Others^†^	9 (100.00)	0.00	0.00	0.00
Father’s occupation	Farmer	206 (71.50)	82 (28.50)	1.00	1.00
	Petty trader	35 (48.60)	37 (51.40)	2.66 (1.57 - 4.50)***	1.21 (0.57 - 2.55)
	Employed	48 (53.30)	42 (46.70)	2.20 (1.35 - 3.58)**	0.61 (0.25 - 1.50)
	Others^††^	5 (100.00)	0.00	0.00	0.00
Monthly expenditure (Eth Birr)	<=300	90 (80.40)	22 (19.60)	1.00	1.00
	301-600	97 (77.60)	28 (22.40)	1.18 (0.63 - 2.21)	1.12 (0.55 - 2.28)
	601-900	40 (51.30)	38 (48.70)	3.89 (2.04 - 7.40)***	3.24 (1.46 - 7.17)**
	901-1200	38 (50.70)	37 (49.30)	3.98 (2.08 - 7.63)***	3.41 (1.56 - 7.43)**
	>1200	29 (44.60)	36 (55.40)	5.08 (2.58 - 9.98)***	4.97 (2.21 - 11.16)***

Significant at *p-value < 0.05, **p-value < 0.01, and ***p-value < 0.001.

^†^(farmer, daily laborer), ^††^(carpenter, barber, daily laborer), ^†††^(government and private).

**2-Unreadiness during menstruation (see Table** 3**: categories)** Even though girls knew disposable sanitary napkins as menstrual soak up, most of them used homemade cloth or underwear. Lack of money, local access and knowledge and skill gap on how to use sanitary napkins were some of their reasons why they didn’t use sanitary napkins. In addition to these some girls didn’t buy sanitary napkins from shops since they were ashamed to do so. From the discussion with female teachers two schools were providing sanitary napkins. However, girls reported that they were hesitant to use the sanitary pads given from schools because male students follow their activities and teasing at them.

*“The teacher had taught and told us, in the gender clubs, to use sanitary napkins. However, since male students follow us while we change menstrual soak up in school and teased at us, we didn’t use sanitary napkins ” (****FGD participant)***

*“I felt ashamed to buy sanitary napkins from the shop and I thought that people might talk about my monthly menstrual period while I ask the sanitary napkins, and thus I didn’t use a sanitary napkin”(****FGD participant)***

Misperception about taking a bath during menstruation days was common. As revealed from the two focus group discussions, girls didn’t take a bath while having their menstruation thinking that bathing might aggravate the bleeding.

*“I had never had a bath while having my periods, as people often say, it might aggravate the bleeding” (****FGD participants)***

### Influence of menstruation on girls academic performance

Most, 308 (90.06%) of the students didn’t feel comfortable when they came to school during menstruation days. Ninety two (20.22%) students had missed exams when exams coincided with their menstruation days. This was because of lack of pads and underwear to manage their menstrual bleeding, severe pain related to menstruation and embarrassment.

Over half, 263 (57.80%) of respondents had perceived that menstruation had affected their academic performance or rank negatively as compared to what they had before their menarche. This was due to lack of their class concentration during their period 209 (79.47%), poor class attendance 72 (27.38%), concentration on pain 52 (19.77%), having class tests during their period and did without concentration (13 students) and didn’t prepare for tests (11 students) due to menstruation related problems. The qualitative study supports the findings from the survey as presented below.

#### 3-Influence of menstruation on girls’ academic performance (see Table 3: categories)

All participants perceived that menstruation had an influence on girls’ academic performance. During menstruation days students didn’t come to school or even if they came, they didn’t attend class attentively thinking of the sudden leakage or the pain associated with menstruation. They didn’t come to school even if they have an exam or didn’t do the test with concentration when menstruation days coincide with exam days. They didn’t stand in front of students to answer questions or to write on the board fearing the sudden leakage of blood and staining of their cloth.

*“Eh… since students didn’t know about menstruation and its management…it might be a cause of school dropout, might have influence on class concentration and class participation. For example, if their menstrual period coincided with school days, they might not come to school until the cycle ends fears of mistreatment of others (particularly male) students. In addition to this, they didn’t communicate the issue with their teachers rather kept it as their own secret or private issue because of fear of embarrassment” (****IDI-teacher)***

*“… During menstruating days, since our attention is disturbed by the thinking of the sudden leakage of menstrual blood and staining of the cloth, we didn’t concentrate our attention in the class. Our attention to education or class is decreased during menstruating days as it is compared with non-menstruating days” (****FGD participant)***

### Influence of menstruation on girls school absenteeism

More than half, 248 (54.51%) of the girls were absent from school during their last menstrual period from one to four days. The mean days of school absenteeism were 2.09 and 2.33 among those who used sanitary napkins and those who didn’t respectively. The main reasons for their absence were; shame and fear of sudden leakage or staining, 204 (82.26%) followed by didn’t have pad, 139 (56.05%) and no private place to manage a menstrual period in their school, 78 (31.45%) (Table 7).

**Table 7:** **Reasons of school absenteeism during menstruation days, Northeast Ethiopia, 2013 (n = 248)**

Reasons (n = 248)	Number	Percent
Shame/fear of leakage/staining	204	82.26
Had no pad to manage period	139	56.05
No private place to manage period at school	78	31.45
Lack of continuous water supply	57	22.98
Pain/discomfort	52	20.97
Lack of disposal system for pads/cloths	21	8.47
Lack of separate bathroom for girls	19	7.66

NB: Multiple responses were possible and the percent is greater than 100%.

The multivariate analysis showed that students who didn’t use disposable sanitary napkins were 5.37 times more likely to be absent from school than their counterparts [AOR (95% C.I): 5.37 (3.02, 9.55)]. School girls who were learning in urban area (Melka Chefe junior secondary school) were 59% less likely to be absent from school during their menstrual period than girls from other schools [AOR (95% C.I): 0.41 (0.18, 0.90)] (Table 8). However, residence, living conditions, parental education and occupation; monthly expenditure and knowledge about menstruation were confounders of school absenteeism.

**Table 8:** **Factors associated with school absenteeism during menstruation days, Northeast Ethiopia, 2013 (n = 455)**

Variables		School absenteeism		Crude odds ratio (95% C.I.)	Adjusted odds ratio (95% C.I.)
		No	Yes		
		n (%)	n (%)		
School	Sirinka	23 (35.90)	41 (64.10)	1.00	1.00
	Kokono	13 (37.10)	22 (62.90)	0.95 (0.40, 2.23)	0.66 (0.25, 1.76)
	Merto	26 (39.40)	40 (60.60)	0.86 (0.42, 1.76)	0.73 (0.32, 1.64)
	Sirinka Gerado	16 (39.00)	25 (61.00)	0.88 (0.39, 1.97)	0.72 (0.28, 1.88)
	Mehal Amba	18 (45.00)	22 (55.00)	0.69 (0.31, 1.53)	0.49 (0.20, 1.20)
	Melka Chefe	60 (57.10)	45 (42.90)	0.42 (0.22, 0. 80)**	0.41 (0.18, 0.90)*
	Mersa	51(49.00)	53 (51.00)	0.58 (0.31, 1.11)	0.68 (0.31, 1.46)
Residence	Urban	116 (52.30)	106 (47.70)	1.00	1.00
	Rural	91 (39.10)	142 (60.90)	1.71 (1.18, 2.48)**	0.95 (0.54, 1.66)
Live with	Both parents	165 (48.50)	175 (51.50)	1.00	1.00
	Mother only	26 (41.30)	37 (58.70)	1.34 (0.78, 2.31)	1.26 (0.68, 2.34)
	Father only	5 (45.50)	6 (54.50)	1.13 (0.34, 3.78)	0.76 (0.20, 2.88)
	Relatives	7 (20.00)	28 (80.00)	3.77 (1.60, 8.87)**	2.39 (0.96, 5.96)
	Husband	4 (66.70)	2 (33.30)	0.47 (0.09, 2.61)	0.62 (0.09, 4.15)
Mother’s education	Illiterate	73 (37.40)	122 (62.60)	1.00	1.00
	Read & write	67 (49.60)	68 (50.40)	0.61 (0.39, 0.95)*	0.98 (0.58, 1.67)
	Primary	35 (52.20)	32 (47.80)	0.55 (0.31, 0.96)*	1.22 (0.60, 2.46)
	Secondary and above	32 (55.20)	26 (44.80)	0.47 (0.27, 0.88)*	1.70 (0.73, 3.98)
Mother’s occupation	House wife	123 (44.10)	156 (55.90)	1.00	1.00
	Petty trader	59 (44.70)	73 (55.30)	0.98 (0.64, 1.48)	1.19 (0.71, 1.98)
	Employed	23 (65.70)	12 (34.30)	0.41 (0.20, 0.86)*	0.55 (0.21, 1.43)
	Others^†^	2 (22.20)	7 (77.80)	2.76 (0.56, 13.52)	1.56 (0.29, 8.39)
Monthly expenditure (Eth Birr)	>1200	33 (50.80)	32 (49.20)	1.00	1.00
	<=300	39 (34.80)	73 (65.20)	1.93 (1.04, 3.60)*	0.90 (0.42, 1.90)
	301-600	51 (40.80)	74 (59.20)	1.50 (0.82, 2.74)	0.81 (0.40, 1.65)
	601-900	43 (55.10)	35 (44.90)	0.84 (0.43, 1.62)	0.61 (0.29, 1.30)
	901-1200	41 (54.70)	34 (45.30)	0.86 (0.44, 1.66)	0.68 (0.32, 1.44)
Knowledge	Poor knowledge	2 (14.30)	12 (85.70)	1.00	1.00
	Fair knowledge	63 (33.70)	124 (66.30)	0.33 (0.07, 1.51)	0.41 (0.08, 2.00)
	Good knowledge	142 (55.90)	112 (44.10)	0.13 (0.03, 0.60)**	0.40 (0.08, 2.03)
Disposable sanitary pad use	Yes	116 (72.00)	45 (28.00)	1.00	1.00
	No	91 (31.00)	203 (69.00)	5.75 (3.76, 8.78)***	5.37 (3.02, 9.55)***

Significant at *p-value < 0.05, **p-value < 0.01, and ***p-value < 0.001.

^†^(farmer, daily laborer).

#### 4-Reasons of school absenteeism (see Table 3: categories)

During menstruation days, some girls didn’t come to school as their schools are not gender friendly, i.e., as their schools lack sanitary facilities to manage their hygiene and due to lack of underwear or sanitary napkins.

*“Eh… in schools, there is nothing totally; i.e., there is no toilet, water for drinking and washing, soap… sanitary disposal facility. Eh… there is no underwear and sanitary napkins available for girls. Similarly… there is no private place where girls can change their underwear or sanitary napkins and manage their hygiene while they are in school. Eh… in rural areas; girls did not have sanitary materials or underwear and lack of sanitary facilities in schools influence female student school absenteeism during menstruation days. Eh… fulfilling these gaps in all schools can enhance girl students’ school attendance and their academic performance too”****IDI-1 (Teacher)***

### Influence of menstruation on school dropout

Regarding school dropout due to menstruation related problems, out of 551 students who had information about menstruation, 136 (24.68%) knew a girl or some girls who had dropped out of school while 140 (25.41%) of school girls reported that they have heard about girls who had dropped out of school. The main causes of school dropout were embarrassment, not having menstrual soak ups, lack of cleanliness and separate toilet and water access in their schools.

Among 455 students who had reached menarche 21 of them were previously dropped out due to menstruation related problems. Their reasons were lack of sanitary napkins and underwear, embarrassed due to menstruation, and lack of cleanliness and separate toilet facilities for girls in their schools. The qualitative study supports the findings from the survey as presented below.

#### 5-Reasons of school dropout (see Table 3: categories)

Previously, some students decided to drop out their education since they didn’t know or learnt about menstruation as well as its management. Embarrassment leads to school dropout when they have their menstruation while in the class suddenly. Following the incident, since other students’ particularly male students didn’t have the knowledge and mistreated them, they dropped out. It was also associated with lack of sanitary material and fear of sudden leakage as they feared the psychological trauma faced when students teased at them, i.e., embarrassment.

*“… Yes menstruation had an influence on my education. One day, when I was in school, the blood suddenly leaked and had stained my cloth and male students teased at me; then after, I went home and decided not to go back to school the next day and even dropped out of school too”****(IDI school-dropout girl)***

*“Yes, even though the students didn’t tell you the exact reason, I know a girl who dropped out of school due to menstruation. … She dropped out of education due to menstruation. She dropped out as other students, particularly male students, teased at her and she was embarrassed while her private issue became public”****IDI-1 (Teacher)***

## Discussion

This study showed that about half the girls in Habru woreda had knowledge regarding menstruation and its hygienic management. However, access and use of sanitary napkins were very low, especially among girls from rural areas, family of lower income, who live with relatives and less educated families. Girls in the study area dropped out of school because of embarrassment as a result of being seen by other students in a blood stained dress usually as a result of having their first menses at school without prior preparation. Absenteeism from school at least for three days per month was common because of lack of sanitary napkins.

In this study above half (51.36%) of the students had good knowledge about menstruation and its management. But there is a knowledge gap in specific areas like why girls have menstruation (cause) and where the blood does during menstruation comes from (origin). This may be related to the low parent to child communication about menstruation and its management too. The reported knowledge is closely related to a study done in Nigeria [5]**.** In contrary to this study finding, a lower knowledge score was obtained in a study done in northwest Nigeria [19]**.** The qualitative data had also documented that girls’ in this study had poor knowledge regarding menstruation and its management. This good knowledge score might be obtained due to a change in time and advancement of educational provisions through different media as compared to the previous time. On the other hand, the documented poor knowledge in the qualitative data might reflect the knowledge gaps; of the quantitative study of female students in specific areas of menstruation and its management.

This study showed that the majority of the girls had experienced different negative reactions when they had seen their first menstruation. The qualitative data also support this fining as girls faced different psychological trauma and had misperceptions about menstruation since they didn’t have prior knowledge about menstruation and its management. These reactions might be due to unawareness of the biological nature as well as unpreparedness leading to soiling up of cloth as they might be teased by others or a reflection of taboos and prejudices in society about menstruation. These negative reactions towards menarche were supported by other studies; they were ashamed [5], irritated or disgusted [4, 23, 24], upset or tensioned [2]**,** scared [4, 23, 24] and felt as guilty [5, 24].

Girls in the study area used pieces of cloths and rags than disposable sanitary napkins as menstrual absorbent. Only 35.38% of girls used disposable sanitary napkins during their last menstrual period. This is lower as it is compared to a study done in Northwest Ethiopia [12] and Addis Ababa [15]. However, in the qualitative data, even if girls want to use disposable sanitary napkins, they didn’t use because of lack of money, feeling ashamed to buy from shops, unavailable in their area and some of them didn’t know how to use it. A study done in Saoner, Nagpur District had supported girls’ reasons for not using disposable sanitary napkins because of lack of knowledge, high cost, unavailability, shyness and mothers’ restriction not to use sanitary napkins and/or disposal problem [25]. The reasons for not using disposable sanitary napkins might be related to different factors. First, it might be related to poor parental/family communication, and educational level or awareness of mothers and/or elder sisters and students themselves about menstruation and its hygienic management. Second, it might be due to lower socio economic status as disposable sanitary napkins might not be locally accessed and available at an affordable/low cost. Third, it might be due to the negative attitudes and perceptions of the community towards menstruation and its management, which may influence buying sanitary napkins from shops. Fourth, in addition to the above mentioned, it might be related to school environment, i.e., students’ awareness, presence of different school clubs like gender clubs, teachers attitude/perception, provision of sanitary napkins and availability of sanitary facilities in schools separated for male and female students.

Disposable sanitary napkins utilization was significantly associated and was higher among urban residence, those who had literate mothers and those from families wealthier (had higher family monthly expenditure). On the other hand it was significantly lower among those living with relatives. This may be because urban girls might have better knowledge; or access to information and/or sanitary napkins as compared to their counterparts where their utilization of disposable sanitary napkins might be higher as compared to their rural counterparts. Educated mothers might teach their daughters and/or might have a monthly budget for sanitary napkins; and a higher family monthly expenditure might include sanitary napkins expense. Therefore, sanitary napkins use among girls from educated mother and families of higher monthly expenditure might be higher. On contrary to these, those who live with relatives might not discuss openly about menstruation and/or relatives might not buy sanitary napkins and thus they might be less likely to use sanitary napkins. In other studies mother’s education [5, 26]**,** residence [25] and socioeconomic status [27] was significantly associated with hygienic management as well as utilization of disposable sanitary napkins.

Over half (54.51%) of the girls had been absent from school during their last menstrual period. This is in contrast to other studies where school absenteeism was lower than this study finding [1, 12–15, 26]. In another study, girls were less likely to attend school (2.40 percentage points) on the days they had their menstruation as compared with other days [28]**.** School absenteeism during menstruation days had also supported by the qualitative data as most students didn’t come to school due to lack of sanitary materials, fearing the sudden leakage of menstrual blood and the mistreatment following the incident. This discrepancy could be due to the type of schools and infrastructure (co-education school), lack of proper knowledge as education could not give in all school curriculums or in different school clubs and lack of sanitary materials. In addition, it could be due to differences in age as the study subjects were younger than other studies, attending primary education, i.e., the probability of being empowered or making decisions as well as facing problems might be different.

School absenteeism was significantly associated and higher among those who didn’t use the disposable sanitary napkins as menstrual absorbent. The mean days of school absenteeism were also higher among those who didn’t use sanitary napkins. On the other hand, school absenteeism was significant and lower among those learning in urban area (Melka Chefe Junior Secondary School). School girls who didn’t use a sanitary napkins might be absent from school fear of sudden leakage as male students might mistreat them.

Menstruation had an influence on adolescent girls’ school performance as over half of the girls reported the perceived effect of menstruation on academic performance. Similar studies had also reported effect of menstruation on girls’ academic performance [11, 15]. The qualitative data explained that during menstruation days students didn’t come to school or even if they came to school; they didn’t attend class attentively thinking about pain if there is or whether the blood leaked suddenly. This might be due to the prevailing knowledge gap or misperceptions of girls; more importantly male students; no school health education program (menstrual hygiene management). It could also relate with the school facility and/or school consultancy services.

Menstruation and its related problems had influenced girl students to drop out their education. In rural Peru, the beliefs and taboos associated with menstruation strongly encouraged girls to remain at home during their period, which contributes to the high rates of school absenteeism, repetition, and school dropout [29]. It is also supported by other studies as most girls had dropped out their education due to menstruation related problems [8, 16]. The qualitative data had also supported this finding as most of the reasons were an embarrassment and lack of menstrual soak ups. These dropouts might be due to poor parental communication regarding sexual and reproductive health issues particularly menstrual management. There might not be anybody who advises them (families, teachers, etc.); lack of sanitary napkins provision or self support club in schools and school facilities might have influence as they were not gender friendly.

### Limitations of the study

Due to its cross-sectional nature of the study, it is difficult to establish causal relationship between the dependent and predicting variables. In the qualitative part of the study, as the study subjects were selected purposively, there might be selection bias. It is also difficult to acquire the exact age of menarche as there may be recall bias and also girls do not report their exact age as there is no birth registration in Ethiopia and girls might tend to underestimate their age. In addition to the above mentioned limitations, being a school based study makes it difficult to get the appropriate denominator for school dropout due to menstruation related problems in the quantitative study.

## Conclusion

The results of this study suggested that girls in the study area were forced to abstain and drop out of school because of lack of disposable sanitary napkins and sanitation facilities at school. The fact that about half of the students did not have the right knowledge about menstruation suggests that adolescent reproductive health needs is neglected in rural areas of Ethiopia and is a gray area for intervention in light of increasing girls’ school enrollment. Thus, in order to realize girls’ empowerment hence gender equality, emphasis should be given for the need of girls’ preparedness for menarche and sanitary disposal facilities at school and at a community level. Increasing mother’s awareness about menstruation and its hygienic management and encouraging daughter-mother communication is justified based on the findings of this study. Including menstruation management in elementary school education could help involvement of boys too. Policy makers should also give special attention towards making schools a comfortable place for girls.

## Authors’ information

Teketo Kassaw earned an MPH, in 2013, from Addis Ababa University, College of Health Sciences, School of Public Health and lecturer at Debre Markos University, College of Medicine and Health Sciences, Public Health department.

Mitike Molla earned a PhD at the Center for International Health at the University of Bergen and Postdoctoral Fellowship at Emory University; Assistant professor in the Behavioral Health Sciences Unit, School of Public Health, College of Health Sciences, Addis Ababa University.
